# Role of plastids and mitochondria in the early development of seedlings in dark growth conditions

**DOI:** 10.3389/fpls.2023.1272822

**Published:** 2023-09-29

**Authors:** Salek Ahmed Sajib, Margot Kandel, Sadia Akter Prity, Cylia Oukacine, Bertrand Gakière, Livia Merendino

**Affiliations:** ^1^ Université Paris-Saclay, CNRS, INRAE, Université Evry, Institute of Plant Sciences Paris-Saclay (IPS2), Gif sur Yvette, France; ^2^ Université Paris-Cité, CNRS, INRAE, Institute of Plant Sciences Paris-Saclay (IPS2), Gif sur Yvette, France

**Keywords:** skotomorphogenesis, etioplasts, mitochondria, development, retrograde control

## Abstract

Establishment of the seedlings is a crucial stage of the plant life cycle. The success of this process is essential for the growth of the mature plant. In Nature, when seeds germinate under the soil, seedlings follow a dark-specific program called skotomorphogenesis, which is characterized by small, non-green cotyledons, long hypocotyl, and an apical hook-protecting meristematic cells. These developmental structures are required for the seedlings to emerge quickly and safely through the soil and gain autotrophy before the complete depletion of seed resources. Due to the lack of photosynthesis during this period, the seed nutrient stocks are the primary energy source for seedling development. The energy is provided by the bioenergetic organelles, mitochondria, and etioplast (plastid in the dark), to the cell in the form of ATP through mitochondrial respiration and etio-respiration processes, respectively. Recent studies suggest that the limitation of the plastidial or mitochondrial gene expression induces a drastic reprogramming of the seedling morphology in the dark. Here, we discuss the dark signaling mechanisms involved during a regular skotomorphogenesis and how the dysfunction of the bioenergetic organelles is perceived by the nucleus leading to developmental changes. We also describe the probable involvement of several plastid retrograde pathways and the interconnection between plastid and mitochondria during seedling development. Understanding the integration mechanisms of organellar signals in the developmental program of seedlings can be utilized in the future for better emergence of crops through the soil.

## Introduction

**Figure 1:**
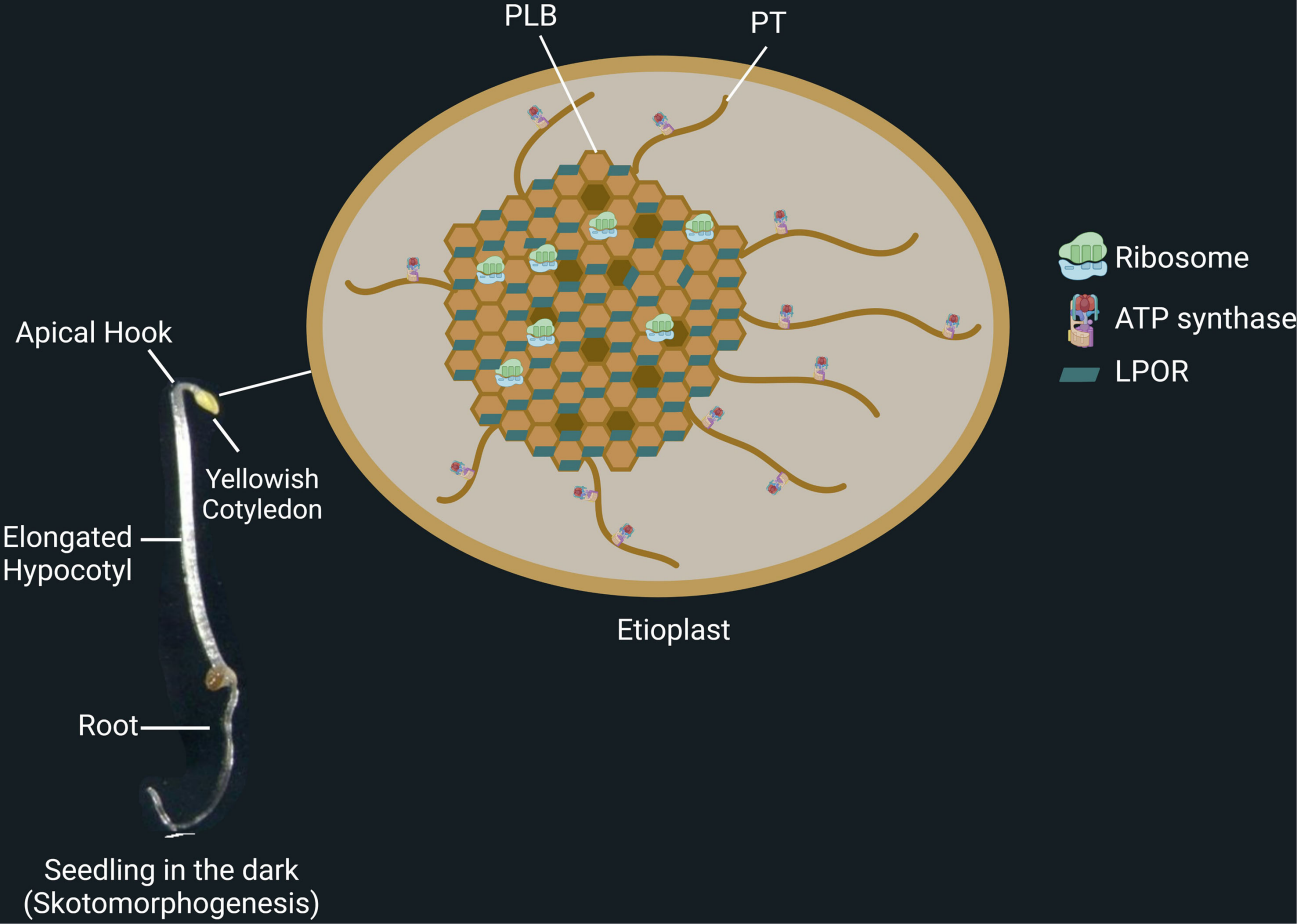
Skotomorphogenesis is the dark-specific developmental program of seedlings. Seedlings growing in darkness present unique structures, including apical hook, long hypocotyl, and simple root architecture. At this stage, the seedlings possess etioplasts, small, round plastids that contain proto-thylakoids (PT), prolamellar bodies (PLB), starch, and plastoglobules. Light-dependent protochlorophyllide oxidoreductase (LPOR) binds protochlorophyllide and is coated on PLB membranes. Ribosomes are found in the PLB network, whereas ATP synthases are on the PT membranes.

The successful establishment of seedlings begins with seed germination and ends when the seedling acquires the ability of photosynthesis (Chory et al., 1989; Chory et al., 1996; Leivar et al., 2008). This stage is crucial for the development and productivity of plants. When the seeds are covered with soil, seed germination and early development of seedlings occur in the dark. In under-ground conditions, the seedlings follow a specific developmental program in the dark, called skotomorphogenesis, and grow toward the light as soon as possible. During skotomorphogenesis, seedlings present a long hypocotyl, a tightly folded apical hook, and non-green cotyledons (**Figure 1**, left panel) (Leivar et al., 2008). While the apical hook protects the apical meristem from friction with the soil, the fast-growing hypocotyl ensures that the young seedlings quickly reach the soil surface to perceive light for photosynthesis and autotrophy before the complete depletion of the storage reserves. Light perception triggers a signaling cascade that enhances the changes required for photomorphogenic development (greening and opening of cotyledons, formation of green true leaves, reprogramming of root architecture, and suppression of specific plant organs as the hypocotyl) (Chory et al., 1989; Leivar et al., 2008; Gommers and Monte, 2018; Hernández‐Verdeja et al., 2020). In parallel with those macroscopic changes, regulation of molecular signaling pathways and gene expression is observed when plants are shifted to light. The expression of photosynthesis-associated nuclear genes (PhANGs) is activated, triggering the early phases of chloroplast differentiation (Dubreuil et al., 2018).

During skotomorphogenesis, the energy required for seedling establishment and emergence from the soil is assured by seed storage reserves and the metabolic functions of the bioenergetic organelles in the cell, plastids, and mitochondria. Plastids and mitochondria are semi-autonomous organelles that contain their own genomes but also depend on nuclear gene expression. Anterograde and retrograde pathways link the bioenergetic organelles with the nucleus. Although the regulation of seedling establishment by light and dark signaling has been extensively studied (Seluzicki et al., 2017; Gommers and Monte, 2018; Deepika et al., 2020; Hernández‐Verdeja et al., 2020), limited number of research reports is available on the function of the bioenergetic organelles during skotomorphogenesis. Therefore, this review focuses on the recent findings on the role of plastids and mitochondria and the involvement of the canonical retrograde pathways with potential new co-regulators in the progression of skotomorphogenesis, especially in the formation and maintenance of the apical hook.

## The signals in darkness

**Figure 2:**
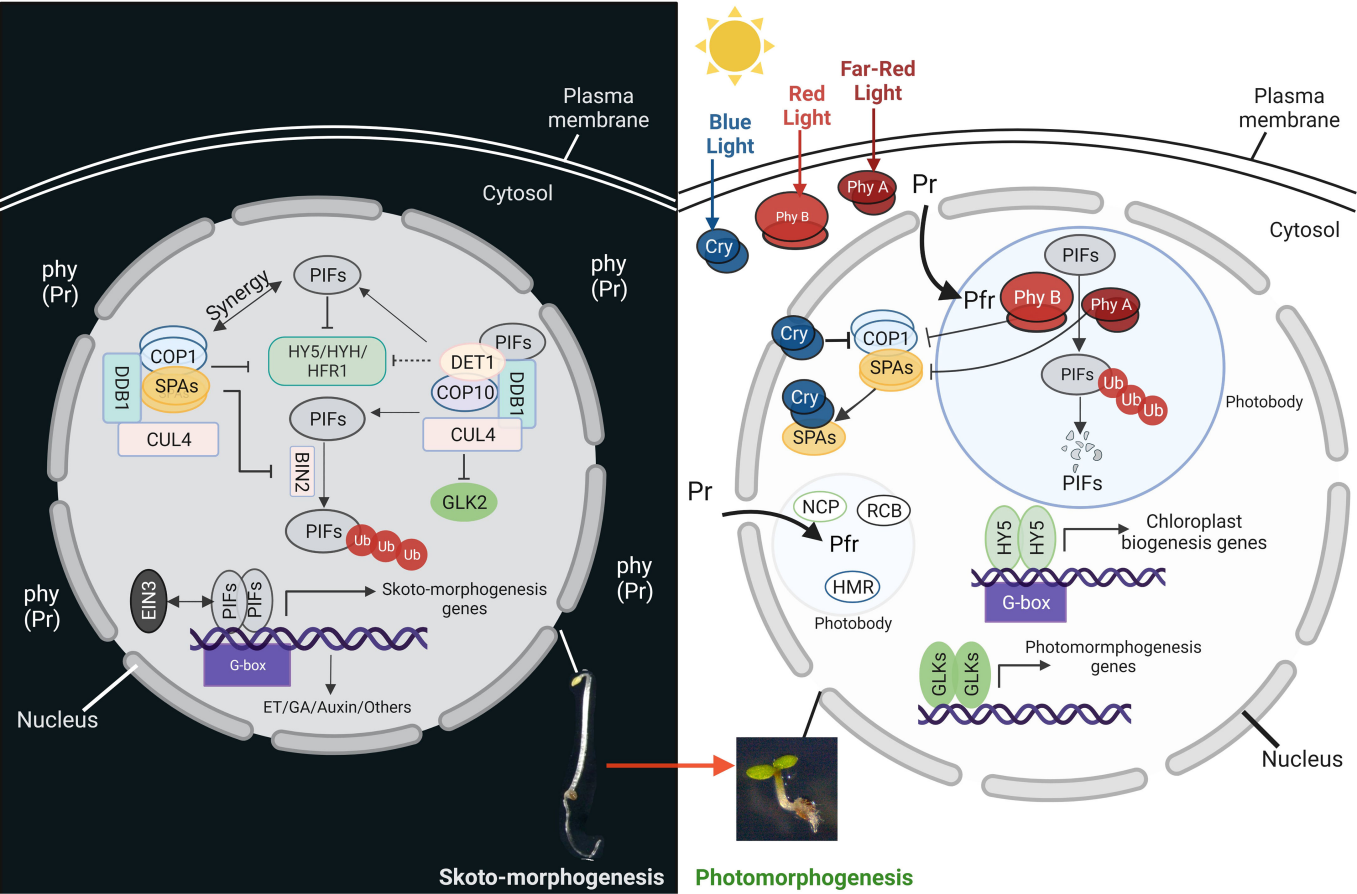
PIFs are the key regulators of skotomorphogenesis. Left panel: during development in the dark, PHYTOCHROME INTERACTING FACTORs (PIFs) act with COP1-SPA complex (CUL4-DDB1-COP1-SPA) and CDD complex (CUL4-DDB1-COP10-DET1) to inhibit the activity of different transcription factors related to chloroplast biogenesis, like HY5, HYH, HFR1 and GLK2. The COP1-SPA complex also acts to stabilize PIFs by inactivating BIN2. The PIFs induce the expression of the genes related to different phytohormone signaling pathways. Right panel: soon after illumination, the cotyledons open and light perception triggers the activation of different photoreceptors. Light-activated cryptochromes (Cry) and phytochromes (from inactive Pr to the active form pfr) interact and/or induce the degradation of the PIFs in photobodies. Moreover, after being activated by the blue light, cryptochromes interfere with the COP1 complexes. All these events release and enhance the activity of different transcription factors like HY5 and GLKs, resulting in the expression of chloroplast biogenesis and function genes.

During skotomorphogenesis, chloroplast biogenesis is blocked by dark-stabilized bHLH transcription factor- PHYTOCHROME INTERACTING FACTORs (PIFs, **Figure 2**). This strategy prevents the accumulation of photosynthetic components that could lead to photodamage when seedlings are shifted from dark to light (Huq et al., 2004; Leivar et al., 2008). Arabidopsis etiolated mutants lacking the four PIFs (*pifq*) resemble plants grown in the light (Leivar et al., 2008). During growth in the dark, PIFs 1, 3, 4, and 5 appear to play dominant roles by regulating more than 2000 genes and promoting rapid elongation hypocotyl growth (Zhang et al., 2013). PIF1 and PIF3 act as master regulators by repressing nuclear genes encoding enzymes of chlorophyll biosynthesis, while PIF4 forms a subsystem with the transcription factor BZR1 that induces brassinosteroid and gibberellic acid synthesis and auxin signaling during skotomorphogenesis (De Lucas et al., 2008; Hornitschek et al., 2012; Oh et al., 2012).

EIN3 is the main transcription factor in the ethylene signaling pathway that is activated in response to mechanical pressure (Liu et al., 2017). Mutations in EIN3 result in early partial chloroplast formation even in the dark and severe photobleaching when exposed to light, indicating the involvement of EIN3 in suppressing etioplast differentiation into the chloroplast. Interestingly, PIF3 is required for EIN3-dependent regulation, and the interplay between the two factors ultimately represses the expression of the genes related to chloroplast formation.

In addition, the transcriptional regulator DELLA, controlled by the phytohormone gibberellin, controls the expression of PIF-regulated genes by inhibiting the binding of PIFs to promoter sequences in the light. The protochlorophyllide oxidoreductase (POR) is one of the target genes of the DELLA factor (Moon et al., 2008; Cheminant et al., 2011; Li et al., 2016).

The repression of photomorphogenesis requires a second group of gene products called CONSTITUTIVE PHOTOMORPHOGENIC/DE-ETIOLATED/FUSCA (COP/DET/FUS) (Seo et al., 2004; Jang et al., 2010; Shi et al., 2015). Two of those factors belong to E3 ubiquitin ligase complexes, COP1-Suppressor of PHYA-105 (SPA) (COP1-SPA) and COP10-DET1-DDB1 (CDD). Their action consists in degrading factors that promote photomorphogenesis and stabilize the PIFs (**Figure 2**) (Seo et al., 2004; Jang et al., 2010; Sheerin et al., 2015; Shi et al., 2015). The COP9 signalosome, or CSN, is the third complex and regulates all CULLIN-RING E3 ligases (CRLs) by deneddylation (Nezames and Deng, 2012). All these complexes are physically connected by a CRL scaffold protein-CULLIN4 (CUL4) (Chen et al., 2010a). COP1-SPA complexes act in synergy with PIFs in the dark and degrade the transcription factors ELONGATED HYPOCOTYL5 (HY5), HY5- HOMOLOG (HYH), and LONG HYPOCOTYL IN FAR -RED1 (HFR1), that promote photomorphogenesis (Hardtke et al., 2000; Jang et al., 2005; Shi et al., 2015). Consequently, *cop1* and *spaQ* (*spa1spa2spa3spa4* quadruple) mutants exhibit the phenotype of premature light-induced development, also known as constitutive photomorphogenic (cop) development, even in the absence of light (Pham et al., 2018). Moreover, COP1 stabilizes PIF3 and EIN3 and promotes the development of cytoplasmic processing bodies (P-bodies) that inhibit the translation of mRNAs involved in chlorophyll production (Jang et al., 2019). Another E3 ligase complex, COP10- DET1-DDB1 (CDD), targets HFR1 and GOLDEN2-LIKE2 (GLK2), a transcription factor required for chloroplast biogenesis, for degradation in the dark (Tang et al., 2016).

The plant switches from heterotrophic to photoautotrophic growth when exposed to light, and the formation of chloroplasts is essential for this switch (Gommers and Monte, 2018). Plants contain various photoreceptors to perceive light and initiate molecular signaling pathways. Five types of photoreceptors have been discovered in Arabidopsis: (a) phytochromes (PHY), (b) cryptochromes (CRY), (c) phototropin, (d) UV RESISTANCE LOCUS 8 (UVR8), and (e) the LOV-F box-cup domain families (Paik and Huq, 2019). When exposed to red light, the inactive form of PHY (Pr) converts to the active form (Pfr) and migrates from the cytosol to the nucleus. The active phytochromes interact with PIFs (PIF1 and PIF3 can interact with PHYA or PHYB, while other PIFs interact mainly with PHYB) and induce phosphorylation and degradation of PIFs by the proteasome in a mechanism that is dependent on photobodies (membraneless organelles containing photoreceptors and factors for transcription, protein sequestration and degradation) (Yamaguchi et al., 1999; Van Buskirk et al., 2012) (**Figure 2**). On the other hand, when cryptochromes perceive blue light, they suppress COP1, which releases and enhances the activity of HY5 and GLK (Ponnu et al., 2019) to control the expression of more than 2000 genes (Lee et al., 2007; Zhang et al., 2011). HY5 and PIFs compete for binding to promoters of photosynthetic genes and, more generally, related to chloroplast biogenesis (Toledo-Ortiz et al., 2014). For example, the synthesis of carotenoids and chlorophyll is negatively controlled by PIFs and positively controlled by HY5.

## Role of bioenergetic organelles during seedling establishment in dark growth conditions.

During skotomorphogenesis, seedlings rely on seed storage and energy metabolism by bioenergetic organelles due to high energy demand and the unavailability of photosynthesis (Gommers and Monte, 2018). Successful establishment of seedlings depends on the proper mobilization of stored nutrients in the seed (Finch-Savage and Bassel, 2016). Various nutrients, including oil (lipid), storage proteins, and starch, are stored in the seed during its maturation and converted into soluble metabolites during germination. Mobilizing the lipid and protein reserves lead to sugar production through gluconeogenesis (Theimer and Rosnitschek, 1978; Sadeghipour and Bhatla, 2003). Through the TCA cycle, mitochondria convert organic matter into chemical energy (ATP) and the reducing power. Then, the reducing force drives the electrons through the KCN-sensitive Electron Transport Chain (ETC) in the inner membrane of mitochondria toward oxygen as the final acceptor. The ETC comprises three major protein complexes with multiple subunits (complexes I, III, and IV) linked by mobile electron carriers. Proton translocation across mitochondrial membranes is used by ATP synthase to generate ATP and is linked to electron transport via this chain (Millar et al., 2011). The TCA cycle can then reuse the oxidized versions of the reducing equivalents generated by ETC as electron sinks. Thus, the TCA cycle and ETC are functionally linked, and any restriction of metabolism in one affects the other (Millar et al., 2011; Meyer et al., 2022).

During skotomorphogenesis, seedlings can also rely on energy metabolism by etioplasts. Although etioplasts are not photosynthetic, they can still provide energy to the organism through etio-respiration. This process enables ATP production by transferring electrons from NAD(P)H of the oxidative pentose phosphate pathway to oxygen, and it is mediated by PTOX and critical factors of the cyclic electron transport as PGR5 and NDH (Kambakam et al., 2016). Etioplasts are round and relatively small plastids with starch, plastoglobules, a prolamellar body (PLB), and proto-thylakoids (PT) (**Figure 1**, right panel) (Pribil et al., 2014; Kambakam et al., 2016; Kowalewska et al., 2019; Floris and Kühlbrandt, 2021). Regular patterns of NADPH, the chlorophyll precursor protochlorophyllide (Pchlide), POR enzymes, together with the lipids of the thylakoid membrane structure, monogalactosyldiacylglycerol (MGDG) and digalactosyldiacylglycerol (DGDG), are all combined to form the structure of a PLB (Pribil et al., 2014; Kowalewska et al., 2019).

Soon after a few hours of illumination, the cotyledons open and light perception triggers the differentiation of proplastids and etioplasts into photosynthetically active chloroplasts in the cotyledon (Gommers and Monte, 2018). Proplastids in the shoot apical meristem are immediately converted into chloroplasts during primary leaf formation. As a result, the expression of PhANGs and Photosynthetic Associated Plastid Genes (PhAPGs) encoding proteins required for chloroplast biogenesis is induced, leading to thylakoid membrane formation and POR-induced chlorophyll production.

## Impact of plastid gene-expression limitation on skotomorphogenesis

Plastids and mitochondria have prokaryotic origins and result from an endosymbiotic event. They are semi-autonomous organelles in that they contain their genomes and their own gene expression machinery, but at the same time, they depend heavily on nuclear gene expression. Most of the protein complexes in the organelles are encoded in two different compartments, the organelle itself and the nucleus.

In *Arabidopsis thaliana*, Nuclear-Encoded RNA Polymerase (NEP) and Plastid-Encoded RNA Polymerase (PEP) are the two types of plastid RNA polymerases involved in plastid transcription (Pfannschmidt et al., 2015). The core complex PEP includes the plastid-encoded proteins rpoA, rpoB, rpoC1, and rpoC2. In addition to the core components, the six nuclear-encoded sigma factors (SIGs) are required for the specificity of the PEP promoter (Lerbs-Mache, 2011). Due to its prokaryotic origin, the activity of PEP can be restricted by antibiotics such as tagetin and rifampicin (Pfannschmidt and Link, 1997). The activity of PEP requires not only the presence of sigma factors but also of PEP-Associated proteins (PAPs) that are nucleus-encoded and plastid-specific (Pfannschmidt and Link, 1994). One of the PAPs, PLASTID REDOX INSENSITIVE 2 (PRIN2), is required to complete PEP activation in a redox-regulated manner (Díaz et al., 2018). In addition, the two nuclear-encoded RNA polymerases, RPOTmp and RPOTp, support the transcription of the plastid genome (Pfannschmidt et al., 2015). NEPs are phage-type polymerases with a single T3/T7 subunit and are, therefore, insensitive to any antibiotic. In contrast to RPOTmp, which is found in mitochondria and plastids (mp), RPOTp is found only in plastids (p). Plastidial genes are divided into three classes based on the RNA polymerase in charge of their transcription. Class I genes are transcribed only by PEP (generally genes coding for subunits of the photosynthetic complexes), class II genes are recognized by both PEP and NEP (e.g., atpB, atpI, ndhB, ndhF, clpP, ycf1), and class III genes are only NEP-dependent (generally housekeeping genes as ycf2, accD, rpoB/C1/C2). As for the transcriptional machinery, most elements of the plastidial translational machinery are encoded in plastids, but factors encoded in the nucleus are also required (Mayfield et al., 2007). Due to the eubacterial nature of the translational machinery (70S ribosomes), various antibiotics, such as lincomycin and spectinomycin, inhibit plastid ribosomal activity.

For a long time, PEP was believed to be active upon light induction to transcribe plastid photosynthetic genes (PhAPGs). In contrast, NEPs were thought to act at early developmental stages to build up the PEP complex and, more in general, the plastid gene expression machinery (Mullet, 1993). The PEP complex was detected in a constitutively active PHYB line and a *pif* quadruple mutant (*pifq*) line, even in dark-growth conditions, but not in light-grown *phy* mutant seedlings (Yoo et al., 2019). These data suggest that PEP complex assembly results from regulation by the two antagonist systems, the promoting light/PHY and the repressive dark/PIFs. Recently, three nuclear-encoded proteins, namely HEMERA (HMR) (Chen et al., 2010b), Regulator of Chloroplast Biogenesis (RCB) (Yoo et al., 2019), and Nuclear Control of PEP Activity (NCP) (Yang et al., 2019), were identified to be localized in plastids as well as in nuclear photobodies. When seedlings lack the activity of these proteins, they exhibit an extensive hypocotyl and albino phenotype with severely impaired chloroplast development in red light. HMR is one of the PEP-associated proteins (PAP5) in plastid nucleoids (Chen et al., 2010b; Galvão et al., 2012). RCB interacts with two other PAPs (THIOREDOXIN Z and FE SUPEROXIDE DISMUTASE3) and shows potential thioredoxin activity *in vitro*, but the role of RCB in the activation or assembly of PEP is still unclear (Yoo et al., 2019). The third PEP-activating protein NCP, an RCB paralog, also contains a thioredoxin domain (Yang et al., 2019).

Even though PEP activity is induced by light, the PEP complex is already entirely built up in the dark, although in smaller amounts than in the green sample (Ji et al., 2021). In addition, the authors show that PEP is active in the dark and responsible for basal transcription of the PhAPGs. These recent data confirm previous studies indicating that both NEPs and PEPs systems are already present in seeds (Demarsy et al., 2006). Seed treatment with the PEP-specific inhibitor tagetin induces a delay in germination, suggesting that PEP is active immediately after seed imbibition in the dark.

**Figure 3:**
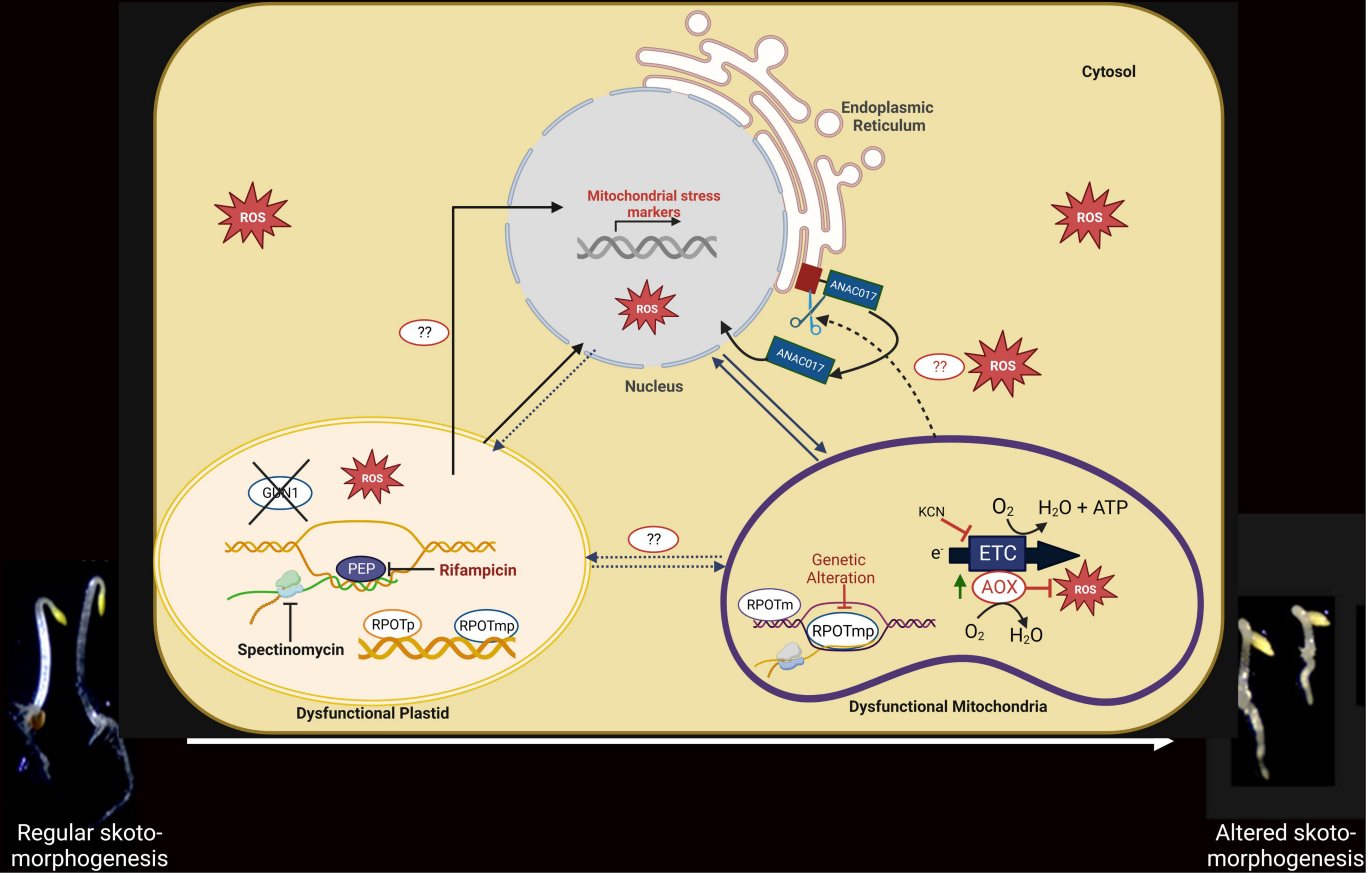
Functional link among PGE limitation, mitochondrial dysfunction, and the developmental response of etiolated seedlings. Under PGE-limited conditions (in the presence of rifampicin/spectinomycin) or under mitochondrial dysfunction (*rpoTmp* mutant), mitochondrial stress marker genes are induced at the transcript level. The expression of the nuclear stress markers is independent of the GUN1 (plastidial) and ANAC017 (mitochondrial) retrograde pathways in the PGE-limited conditions but is dependent on the ANAC017 retrograde pathway in the mitochondrial dysfunction condition. In mitochondrial stress conditions, the ER-localized ANAC017 proteins are cleaved and released for migration into the nucleus and transcriptional regulation. Under both plastidial and mitochondrial dysfunctional conditions, expression of AOX is increased at the mRNA and protein levels; consequently, the AOX-dependent respiratory capacity is also induced. Under PGE-limited conditions, ROS is significantly induced, particularly in the absence of AOX. A developmental response (twisting phenotype) that requires functional AOX is induced as an ultimate effect in both organellar dysfunction conditions. Whether the developmental response in PGE-limited conditions is induced directly by plastid signals or via mitochondria by AOX-generated signals is still a matter of investigation.

A recent study demonstrates that the limitation of plastid transcription or translation by treatment with rifampicin or spectinomycin, respectively, causes an excessive bending of the apical hook (twisting) in etiolated seedlings (**Figure 3**) (Sajib et al., 2023). Furthermore, RPOTp RNA polymerase mutants displayed the same excessive bending of the apical hook, indicating that the twisting phenotype is a specific response to PGE limitation and not the consequence of the pleiotropic effects of antibiotics. In addition, seedlings treated with rifampicin showed changed mitochondrial metabolism, elevated levels of ROS, and enhanced capacity of mitochondrial alternative oxidases to consume oxygen, revealing a potential communication between plastids and mitochondria. Dark-grown WT Arabidopsis seedlings treated with rifampicin showed increased levels of several TCA and glycolysis intermediates and a significant shift in amino acid metabolism, demonstrating that PEP transcription limitation can affect mitochondrial metabolism. Modifications in mitochondrial metabolism may result from communication and signal exchange between plastids and mitochondria, and the signals between the organelles are perhaps transferred via physical connections or through the nucleus. Moreover, nuclear marker genes for mitochondrial stress as *AT12CYS* and *AOX1A*, are highly expressed in the RIF-treated seedlings. These data suggest that the nucleus receives mitochondrial dysfunctional signals when PGE is limited.

## Impact of mitochondrial dysfunctioning in skotomorphogenesis

Like the plastids, due to their prokaryotic and endosymbiotic origin, mitochondria also act as cellular semi-autonomous organelles with their own genome and gene-expression machinery but depend on the nucleus for most protein expression (He et al., 2023). Two phage-like NEP RNA polymerases-RPOTm found only in mitochondria, and RPOTmp, found in both plastids and mitochondria- are responsible for the transcription of the mitochondrial genome (Hedtke et al., 1997; Hedtke et al., 2000). Since they are phage-like enzymes, they are resistant to antibiotics. On the other hand, they contain prokaryotic ribosomes that are sensitive to antibiotics. However, nucleus-encoded Mitochondrial Ribosomal Proteins (MRP) are required for translation of mitochondrial transcripts. During development in the dark, the *rpoTmp* and *mrpL1* mutants and other cytochrome c oxidase (COX)-dependent respiratory mutants (*rug3*, *atphb3*) show significant reprogramming of Arabidopsis seedling architecture with a shortened hypocotyl and/or increased apical hook bending (twist) (Wang and Auwerx, 2017; Merendino et al., 2020), indicating that mitochondrial dysfunctioning at the level of gene expression and respiration impact on skotomorphogenesis (**Figure 3**).

Interestingly, AOX, the ubiquinol oxidase that can prevent the excessive reduction of the respiratory ETC and the formation of harmful reactive oxygen species (ROS), is also activated in the *rpoTmp* mutant seedlings. AOX acts by substracting electrons to the ETC and reducing oxygen. Apical twisting of *rpoTmp* plants is reduced when AOX is blocked with the inhibitor salicylhydroxamic acid (SHAM), while genetic deletion of the major isoform of AOX in the dark, AOX1a (*rpoTmp/aox1a*), resulted in seedlings without apical hook twisting (Merendino et al., 2020). These results suggest that the AOX enzyme integrates signals about mitochondrial functional status into the seedling developmental program. Recently, it was reported that in response to mitochondrial stress, proteolytic cleavage of the mitochondrial retrograde transcription factor ANAC017 occurs, which leaves the endoplasmic reticulum (ER) and moves to the nucleus. There, it can influence the expression of the AOX1a gene, leading to the accumulation of AOX proteins, an increase in their enzymatic capacity, and the promotion of hook-bending exaggeration (Van Aken et al., 2016; Meng et al., 2019; Kacprzak et al., 2020; Merendino et al., 2020). Importantly, genetic disruption of ANAC017 impairs twisting in *rpoTmp* mutant seedlings (Merendino et al., 2020). All these results underline the involvement of ANAC017-dependent mitochondrial retrograde signaling and AOX in the skotomorphogenesis regulation (Merendino et al., 2020). The nature of the AOX-generated signals that lead to skoto-morphogenic reprogramming is still obscure.

## The link between plastids and the nucleus

Plastids are known to be linked to the nucleus through signaling pathways (Pogson et al., 2008; Pfannschmidt, 2010; Hernández-Verdeja and Strand, 2018). Nuclear gene expression changes in response to the functional state of plastids, and in turn, their function is either enhanced or limited. The nucleus can regulate organellar biogenesis and function through anterograde signaling, while organelles can communicate their developmental and functional status to the nucleus through retrograde signaling. In addition, plastids are connected to mitochondria through different metabolic pathways (Eisenhut et al., 2019; Smith et al., 2019; Medeiros et al., 2021). Therefore, effective communication between plastids, the nucleus, and mitochondria is essential for optimal organellar function. Upon exposure to light, phytochromes are activated, and photomorphogenesis is induced. The phytochrome-dependent light signaling pathway is integrated with the retrograde signaling from plastids to modulate the expression of PhANGs. In the presence of excess light, the constraining conditions are sensed by the cell through the damaged plastid, and plastid stress signals are sent to reduce the light induction of the PhANGs (Martin et al., 2016). In this way, the plastid is the light sensor of the cell, and the retrograde signaling pathway allows plants to adapt to the environment.

Retrograde signals are categorized as either biogenic or operational, depending respectively on whether they are produced during organelle biogenesis or by mature organelles in response to developmental signals and environmental stimuli (Chamovitz et al., 1991; Gray, 1995). Various chemical inhibitors are used to identify these plastid signals, such as norflurazon (NF), a potent inhibitor of carotenoid biosynthesis, or several antibiotics targeting the plastid gene expression machinery of prokaryotic origin. NF inhibits the phytoene desaturase, the crucial input enzyme of carotenoid biosynthesis (Chamovitz et al., 1991). Seedlings treated with NF exhibit white cotyledons instead of yellowish in the dark or green in the light indicating successful suppression of carotenoid production. On the other hand, treatments with tagetin and rifampicin effectively inhibit prokaryotic-type polymerase transcription PEP (Rapp and Mullet, 1991; Pfannschmidt and Link, 1997), and antibiotics such as chloramphenicol, spectinomycin, or lincomycin (Lin) restrict plastid translation (Oelmüller and Mohr, 1986; Oelmüller et al., 1986; Gray, 1995).

**Figure 4:**
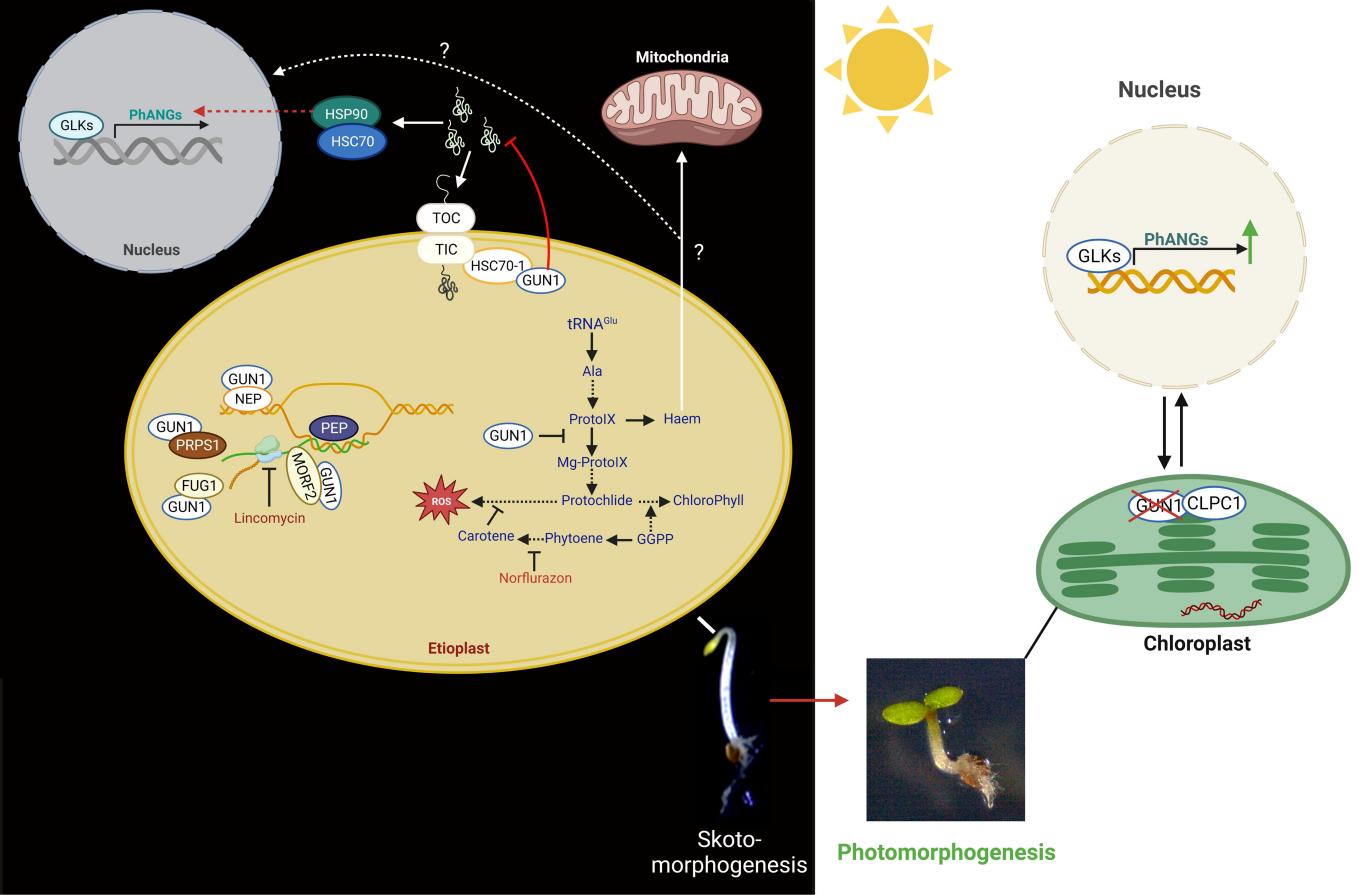
Functions of GUN1 in plastid biology and retrograde control of nuclear gene expression. Left panel: in the dark, by interacting with NEP, MORF2, FUG1, and PRPS1, GUN1 controls plastid gene expression (PGE). GUN1 is also involved in plastid protein import by interacting with HSC70-1 and the TIC/TOC complex. When GUN1 is not present, preproteins and, consequently, cytosolic chaperones (HSP90 and HSP70) are accumulated in the cytosol. The HSP90 is thought to be involved in the induction of the expression of the PhANGs, either by repressing negative or activating positive transcription factors. GUN1 can also bind the haem and the enzymes of the TBP, such as the D-subunit of Mg-chelatase (CHLD) and FC1, and thus may affect the flux through the TBP. Moreover, GUN1-mediated signals might influence the expression of GLK1 and GLK2, which modifies the expression of the PhANGs and the essential TBP genes. Most of the molecular functions of GUN1 were identified using the plastid translation inhibitor lincomycin (LIN) or the inhibitor of the carotenoid biosynthetic pathway, norflurazon (NF). Right panel: in the light, levels of GUN1 proteins are strongly decreased by the action of the plastid CLPC1 protease, leading to the expression induction of PhANGs.

When plastid activity is disrupted, one of the most critical indicators of plastid retrograde signaling is a reduction in the expression of PhANGs, including light-harvesting chlorophyll A/B binding protein (LHCB) and RBCS (Oelmüller and Mohr, 1986; Oelmüller et al., 1986; Gray, 1995; Wu and Bock, 2021). Genome uncoupled (*gun*) mutants exhibiting impaired plastid-to-nucleus communication were discovered due to this particular molecular phenotype. In the presence of the *gun* mutations, LHCBs genes were not repressed in response to norflurazon (NF) or Lin (Susek et al., 1993). Among six GUN proteins, five (GUN2-6) belong to the tetrapyrrole biosynthetic pathway (TBP) and control branching pathways downstream of protoporphyrin IX, whereas GUN1 is not an enzyme of TBP (Wu and Bock, 2021). GUN1 is a plastid-localized, nuclear-encoded protein with pentatricopeptide repeat (PPR) and small MutS-related (SMR) domains involved in RNA binding and DNA repair, respectively (Tadini et al., 2016; Tadini et al., 2020). It has been suggested that GUN1 integrates several signals associated with plastid dysfunction, particularly those generated by inhibitors of plastid differentiation, such as norflurazon or lincomycin, or defective TBP (Sullivan and Gray, 1999; Koussevitzky et al., 2007; Tadini et al., 2016; Tadini et al., 2020). GUN1 was shown to interact with nuclear-encoded plastid RNA polymerase (NEP), multiple organellar RNA editing factor 2 (MORF2), a GUN1 interactor (FUG1), and plastid ribosomal protein S1 (PRPS1) (Tadini et al., 2016; Zhao et al., 2019) (**Figure 4**). These compounds promote translation initiation, protein import into the plastid, proper RNA editing of specific plastid-encoded transcripts, and NEP transcriptional activity. By interacting with those factors, GUN1 is supposed to modulate PGE and protein homeostasis (Tadini et al., 2016).

In response to lincomycin treatment, GUN1 regulates the expression of the genes encoding the chloroplast subunits TIC (TRANSLOCON AT THE INNER ENVELOPE MEMBRANE OF CHLOROPLASTS) and TOC (TRANSLOCON AT THE OUTER ENVELOPE MEMBRANE OF CHLOROPLASTS) to link the malfunctioning chloroplast protein synthesis with the impaired protein import ability (Tadini et al., 2020). In addition, GUN1 interacts with HEAT SHOCK COGNATE PROTEIN 70-1 (HSC70-1), which is associated with the TIC complex and facilitates the import of plastid proteins (Wu et al., 2019; Tadini et al., 2020; Wu and Bock, 2021). When GUN1 is absent, unimported preproteins are prominent, and the degree of protein ubiquitination in the cytosol is much higher (Tadini et al., 2020; Wu and Bock, 2021). As a result, the chaperones HEAT SHOCK PROTEIN 90 (HSP90), HEAT SHOCK PROTEIN 70 (HSP70), and HEAT SHOCK COGNATE PROTEIN 70-4 (HSC70-4) are found to accumulate more in the cytosol (Wu et al., 2018). Their accumulation ultimately results in the induction of PhANGs expression (Wu et al., 2018; Tadini et al., 2020). The authors conclude that the nucleus might sense the accumulation of pre-proteins as a stress plastid signal that acts by either activating positive or inhibiting negative transcriptional regulators in the nucleus for the expression of PhANGs (Wu et al., 2018).

The HSP90 chaperone is a significant player also in the GUN5-dependent retrograde signaling pathway, necessary for transmitting retrograde signals from both PGE and TBP (Wu et al., 2018). GUN1 can directly bind tetrapyrroles, confirming their possible function in mediating retrograde signaling, and interact with TBP enzymes to alter flux along the system. Protochlorophyllide (Pchlide), a precursor of phototoxic chlorophyll, has been linked to GUN1 (Shimizu et al., 2019), and this impact may account for the delayed greening and poor survival of *gun1* seedlings during the period of etiolation to de-etiolation (Susek et al., 1993).

A recent study suggests that GUN1 is already present in both proplastids and etioplasts, and its protein concentration decreases in the presence of light and with the progression of chloroplast development (Hernández‐Verdeja et al., 2022). GUN1 plays a protective role in dark-grown seedlings by limiting the expression of transcriptional regulators of genes involved in photomorphogenesis, particularly in photosynthesis. In etiolated seedlings, GUN1 controls the expression of the transcription factors PIFs, BZR1, and BES1, as well as GLK1, and can also repress the GLK1-induced expression of PhANGs. As chloroplast development progresses and the potential risk of oxidative damage decreases, GUN1 is degraded, allowing full expression of PhANGs (Wu et al., 2018; Hernández‐Verdeja et al., 2022). Thus, GUN1 could serve as a protective agent during the critical step of seedling emergence from darkness by repressing PhANG expression and chloroplast formation.

## Role of retrograde signaling pathways in the regulation of skotomorphogenesis by PGE limitation

**Figure 5:**
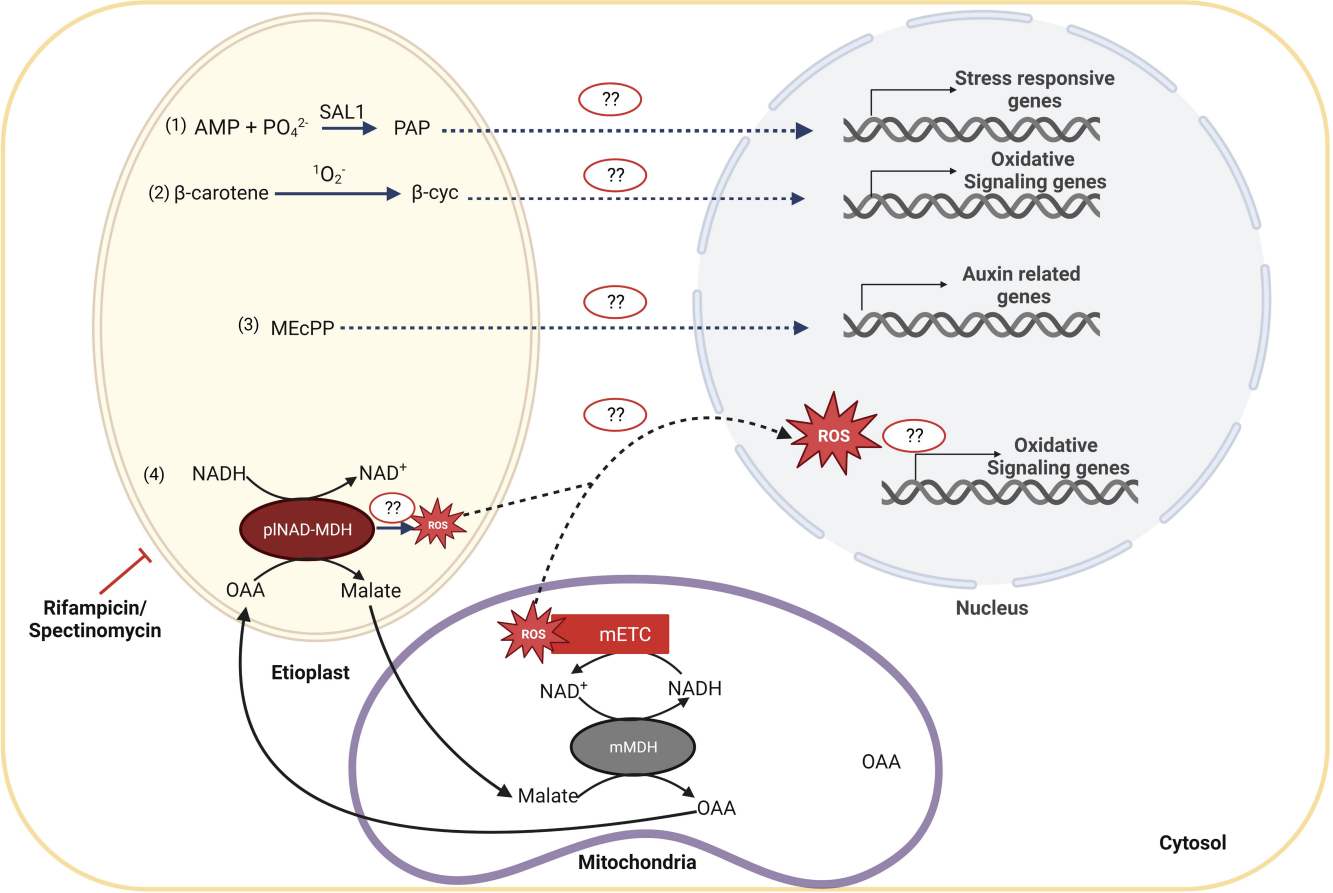
Candidates for retrograde signaling pathways acting during PGE-limited conditions during shoto-morphogenesis. In PGE-limited conditions, distinct plastid metabolites might accumulate and activate the corresponding signaling pathway, as (1) 3′-phosphoadenosine 5′-phosphate (PAP); (2) β-cycloidal (β-cyc), that is the product of the cleavage of β-carotene by singlet oxygen and results in the oxidative signaling, (3) the 2-C-methyl-D-erythosphate (MEcPP) that is required for regulation of auxin-related genes, and (4) malate. It has been proposed that plNAD-MDH reduces NADH excess levels in plastids by converting oxaloacetate (OAA) into malate that is then exported to the cytoplasm to be imported into the mitochondria. In mitochondria, malate is converted back to OAA by mMDH, leading to the production of NADH. NADH can provide electrons to the electron transport chain and, if in excess, can ultimately lead to the production of mitochondrial reactive oxygen species (ROS). ROS can further act as a signaling molecule to modulate nuclear gene expression and, at last, to define the structure of the seedling.

How plastid signals are perceived by the nucleus in etiolated seedlings was never deeply investigated until the impact of the limitation of plastid gene expression (PGE) was observed on skotomorphogenesis (Sajib et al., 2023). The authors could observe that the alteration of nuclear expression and the developmental response induced by rifampicin treatment in dark-developing seedlings proceed independently of the classical GUN1-dependent organelle retrograde pathways. Because the PGE limitation was responsible for mitochondrial perturbation, the authors also tested the involvement of the ANAC017-dependent mitochondrial retrograde pathway in the molecular and developmental response to rifampicin treatment. Mutant *gun1* and *anac017* seedlings showed a twisting phenotype and induction of mitochondrial stress marker genes upon treatment with rifampicin, indicating the existence of alternative retrograde pathways than GUN1 and ANAC017 in PGE-limited dark-grown seedlings. Interestingly, a GUN1-independent RS pathway, activating the ethylene signaling pathway, was already described in photomorphogenesis (Gommers et al., 2021). Previously, it was suggested that other plastidial metabolites may be linked to retrograde signaling during PGE-limited conditions, such as 3′-phosphoadenosine 5′-phosphate (PAP), β-cycloidal (β-cyc), 2-C-methyl-D-erythritol 2,4-cyclodiphosphate (MEcPP) and reactive oxygen species (ROS) (de Souza et al., 2017) (**Figure 5**).

PAP is a byproduct of sulfur assimilation reactions, which occur in all organisms throughout all spheres of life. Production of 3′-phosphoadenosine 5′-phosphosulfate, a precursor of PAP, is dependent on ATP levels. Cellular redox state and ROS levels influence the production and availability of ATP and, consequently, of PAP, which becomes an indicator of stress (Estavillo et al., 2011). Several stresses, including drought and excessive light, trigger the accumulation of PAP and subsequent activation of nuclear abiotic-stress-responsive genes. As a result, mutant plants with high levels of PAP exhibit more significant expression of genes that respond to abiotic stress and physically indicate increased tolerance to drought stress. Although PAP can be a signal molecule to control stress responses, its function as a classic plastid-to-nucleus retrograde signal has been debated (Xiao et al., 2013). Further research will be required to determine if PAP levels can affect the molecular and developmental response in plastid gene expression-limited conditions during skotomorphogenesis.

On the other hand, carotenoids are plastidial isoprenoid pigments that are essential for plants to survive (Sun et al., 2018). Etioplasts also produce carotenoid pigments, responsible for the yellowish color of cotyledons in the absence of chlorophyll in etiolated seedlings, despite chromoplasts and chloroplasts also having significant amounts of them (Rodríguez-Villalón et al., 2009). β-carotene is cleaved by singlet oxygen (^1^O_2_^-^), which induces the increase of β-cyc (Ramel et al., 2012). The β-cyc, a volatile and lipid-soluble compound, can cross the lipid membranes and transfer information from the plastid (Lv et al., 2015). However, the specific mechanism of β-cyc activity is still unknown. Therefore, it might be another potential candidate of interest if, under PGE-limited conditions, singlet oxygen accumulates and β-cyc can induce nuclear gene expression and consequent developmental reprogramming.

Moreover, 2-C-Methyl-D-erythritol 2,4-cyclodiphosphate (MEcPP), a plastidial stress-induced signaling molecule, is a mediator in the isoprenoid biosynthesis pathway as well as a retrograde signal (Xiao et al., 2012; Walley et al., 2015). The exact signaling mechanisms are still unknown, even though studies have shown that modulation of MEcPP levels in response to stress signals induces the expression of stress-responsive genes such as hydroperoxide lyase (HPL) and ICS1, resulting in increased salicylic acid levels. Recent studies in bacteria indicate that MEcPP might act through chromatin remodeling by directly disrupting histone H1-like (Hc1) protein interactions with DNA (Grieshaber et al., 2004; Grieshaber et al., 2006). The MEcPP pathway is involved in auxin control (Jiang et al., 2018), and since auxin regulates the development of apical hooks (Abbas et al., 2013), it may be interesting to find out if this pathway also controls skotomorphogenesis.

Reactive oxygen species (ROS), which comprise singlet oxygen, superoxide (O_2_^-^), and hydrogen peroxide (H_2_O_2_), play a crucial role in controlling the different metabolic and molecular processes of plant development. ROS levels can hinder seed germination and other stages of plant growth if they are too low or too high (Bailly et al., 2008). The mitochondrial-localized alternate oxidase (AOX) enzyme participates in the regulation of ROS and nitric oxide (NO) homeostasis by limiting the excessive reduction of the mitochondrial ETC (Vanlerberghe, 2013). A significant amount of ROS was found to be induced in the rifampicin-treated PGE-limited WT seedlings and even higher in *aox1a* mutant seedlings, which showed an inability to exhibit the twisting phenotype (Sajib et al., 2023). These data indicate that, in the presence of PGE dysfunction, ROS might influence the seedling architecture in coordination with AOX. Identifying the cellular production site of the rifampicin-induced ROS using organellar-specific biosensors would clarify the retrograde mechanisms acting during skotomorphogenesis, as the previous report did not aim to determine the ROS accumulation at a subcellular level. Additionally, although several studies have shown that ROS generation can play a significant role in retrograde signaling during seed germination, how ROS might cause a particular transcriptome signature that affects seedling developmental programs in the dark is still an open question (Bailly and Merendino, 2021; Jurdak et al., 2021).

Recent research has linked the transport of malate among distinct cellular compartments to the interaction between plastidial and mitochondrial metabolism (Zhao et al., 2018). The plastidial NAD-dependent malate dehydrogenase (plNAD-MDH) is a green and non-green plastid component that consumes an excess amount of NADH in the plastid by converting oxaloacetate (OAA) in malate (Berkemeyer et al., 1998). Malate is then transported out of the plastid into the cytoplasm through the dicarboxylate transporter 1 (DiT1) and then imported again into mitochondria. In mitochondria, malate is converted back to OAA together with the regeneration of NADH that, by providing electrons to the electron transport chain, can induce the production of mitochondrial ROS and over-expression of AOX (Zhao et al., 2018; Luo et al., 2019; Selinski and Scheibe, 2019). Malate transport thereby contributes to the export of reducing equivalents from the plastid to the mitochondria. Since the previous study reported the induction of ROS and malate in the dark-developing seedling in response to rifampicin-induced plastid dysfunction (Sajib et al., 2023), it might be an exciting area to investigate the connections among the transport of malate, mROS, and AOX with the skotomorphogenesis control under PGE limitation conditions.

## Conclusion

In nature, seeds covered by litter or soil because of animal, atmospheric agents, or human action will germinate underground, adopting a dark-specific developmental program. In agriculture, farm machinery causes soil compaction that affects seedling emergence/root penetration and O_2_ diffusion (hypoxia), especially when soil water is saturated upon heavy rainfalls after long periods of drought. Future research can investigate whether the cellsuse mitochondria and plastids not only as energy suppliers for the developmental process but also as sensors of environmental parameters. It will be of great value to explore if energy-related processes such as etio-respiration and mitochondrial oxidative phosphorylation serve to define the oxic state of the cell allowing seedlings to translate the organellar hypoxic stress as a sign of underground conditions. Integration of developmental signals with retrograde pathways that inform the nucleus of the functional state of the cellular bioenergetic organelles will permit an efficient adaptation of the developmental program of the seedlings to the environmental conditions. Consequently, a complete understanding of the dark signaling integration and the involvement of inter-organellar communication during the development mechanisms need to be explored. Set up of genetic screenings, usage of genetically encoded biosensors and microsensors which can measure the O_2_ content in the soil will contribute in defining novel signaling pathways connecting bioenergetic organellar functional state with seedling developmental program. Thus, in the future, it will be worthwhile transferring this knowledge from *Arabidopsis* to crops such as peas and tomatoes to enhance germination and survival rates in the fields, especially in adverse environmental conditions.

## Author contributions

LM: Funding acquisition, Supervision, Writing – review & editing. SS: Writing – original draft, Writing – review & editing. MK: Writing – original draft. SP: Writing – original draft. CO: Writing – original draft. BG: Writing – review & editing.
